# *PPP5C* pathogenic variant identified: a potential key to gaining insight into developmental and epileptic encephalopathy?

**DOI:** 10.1186/s40348-025-00191-3

**Published:** 2025-04-02

**Authors:** Raffaele Falsaperla, Annamaria Sapuppo, Xena Giada Pappalardo, Roberta Rizzo, Roberta Rocca, Gaia Fusto, Silvia Marino, Vincenzo Sortino, Lucia Saccuzzo, Martino Ruggieri, Marco Fichera

**Affiliations:** 1 Department of Medical Science-Pediatrics, University of Ferrara, Ferrara, 44124 Italy; 2 Unit of Pediatrics and Pediatric Emergency Department, Azienda Ospedaliero-Universitaria Policlinico “Rodolico-San Marco”, San Marco Hospital, Catania, 95121 Italy; 3 Department of Biomedical and Biotechnological Sciences (BIOMETEC), University of Catania, Catania, 95123 Italy; 4 National Council of Research, Institute for Research and Biomedical Innovation (IRIB), Unit of Catania, Catania, 95126 Italy; 5 Postgraduate Training Program in Pediatrics, Department of Clinical and Experimental Medicine, University of Catania, Catania, 95123 Italy; 6 Department of Biomedical and Biotechnological Sciences, Section of Clinical Biochemistry and Medical Genetics, University of Catania, via Santa Sofia, Catania, 95123 Italy; 7 Unit of Pediatric Clinic, Department of Clinica and Experimental Medicine, University of Catania, Catania, Italy; 8 Research Unit of Rare Diseases and Neurodevelopmental Disorders, Oasi Research Institute-IRCCS, via Conte Ruggero 73, Troina, 94018 Italy

**Keywords:** *PPP5C* (protein phosphatase 5 catalytic subunit), Developmental disorders, Status epilepticus, Developmental and epileptic encephalopathy (DEE)

## Abstract

**Background:**

Emerging evidence suggesting a possible link between the *PPP5C* gene (protein phosphatase 5 catalytic subunit; OMIM#600658) and developmental and epileptic encephalopathy (DEE, OMIM#308350), although the clinical significance of pathogenic variants in this gene remains unclear. *PPP5C* is a member of the protein phosphatase catalytic subunit family, which is involved in various signaling pathways governing cell growth, differentiation, and responses to hormonal signals or cellular stress. To date, only one case with a *PPP5C* variant has been reported, associated with a severe neurological phenotype, including microcephaly, failure to thrive, and early-onset seizures.

**Results:**

We report a 12-year-old girl affected by epilepsy and learning disorders. At the age of five, she presented convulsive status epilepticus with respiratory failure at onset and she started anticonvulsant therapy with Levetiracetam with a significant improvement. Genetic analysis revealed a de novo heterozygous missense variant of *PPP5C* gene (c.202 C > T: *p.Arg68Cys*), which had not been previously described in the literature.

**Conclusion:**

This case expands the phenotypic spectrum associated with *PPP5C* variants, highlighting the potential role of this gene inneurological disorders. Our findings may provide some valuable insights into the spectrum of phenotypic manifestations linked to this gene less investigated in neuropediatrics.

## Background

New findings indicate a potential association between the *PPP5C* (protein phosphatase 5 catalytic subunit) gene (OMIM# 600658) and developmental and epileptic encephalopathy (DEE, OMIM#308350) [1]. As a phosphatase, *PPP5C* regulates the reversible phosphorylation of various proteins, influencing the activation or inhibition of numerous signaling pathways, including those involved in cell growth, differentiation, and responses to hormonal signals or cellular stress [2, 3]. The mRNA expression of *PPP5C* is widespread throughout the body based on Uniprot/SwissProt data (https://www.uniprot.org), and abundant in the mammalian brain according to Tissue Expression Database by Jersen Lab (https://tissues.jensenlab.org). This makes *PPP5C* a potential drug target in several diseases, including obesity, cancer, and Alzheimer’s disease [4]. Transcriptomic levels are also found to be significant in embryonic tissues and stem cells in LifeMap Discovery database (https://discovery.lifemapsc.com/). Despite evidence of tissue expression in both the developing and adult central nervous systems (CNS), the contribution of this gene in the neurological mechanisms underlying DEE has not yet been confirmed [1].

In this report, we present a 12-year-old girl affected by epilepsy and learning disorders who carries a de novo variant in the *PPP5C* gene, not previously described. This is only the second case report, after Fielder et al. (2022) [1], analyzing the potential role of *PPP5C* in neurodevelopment and learning disabilities.

## Results

The proband is a 12 years old girl. Third child of non-consanguineous parents, born via spontaneous delivery at 40 weeks of gestation; birth weight 4200 g (95th centile; +1,6 SD). At birth, perinatal asphyxia was reported. Neonatal period was uneventful. She showed normal acquisition of developmental milestones during infancy.

**Fig. 1:**
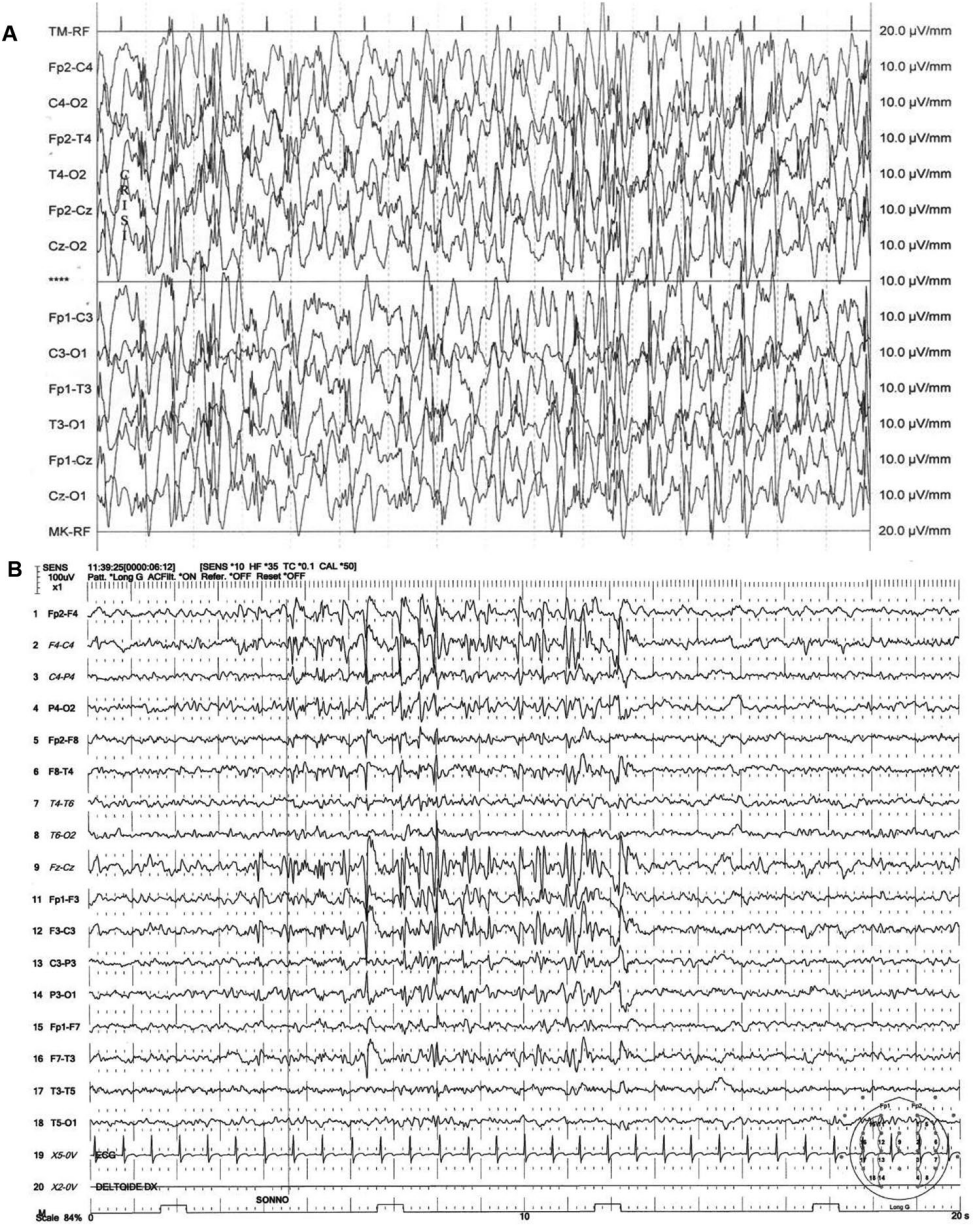
**A**-**B**. EEG of the present patient at the onset during status epilepticus (**A**) and at the age of 12 y.o. (**B**) Standard 10-20 montage. Sens 100 uV. Duration 20 seconds. EEG electrodes: Z: Midline: FZ: Midline Frontal; CZ: Midline Central; PZ: Midline Parietal; OZ: Midline Occipital. Even numbers, right hemisphere locations; odd numbers, left hemisphere locations: Fp: Frontopolar; F: Frontal; C: Central; T: Temporal; P: Parietal; O: Occipital. ECG: Electrocardiogram

**Fig. 2:**
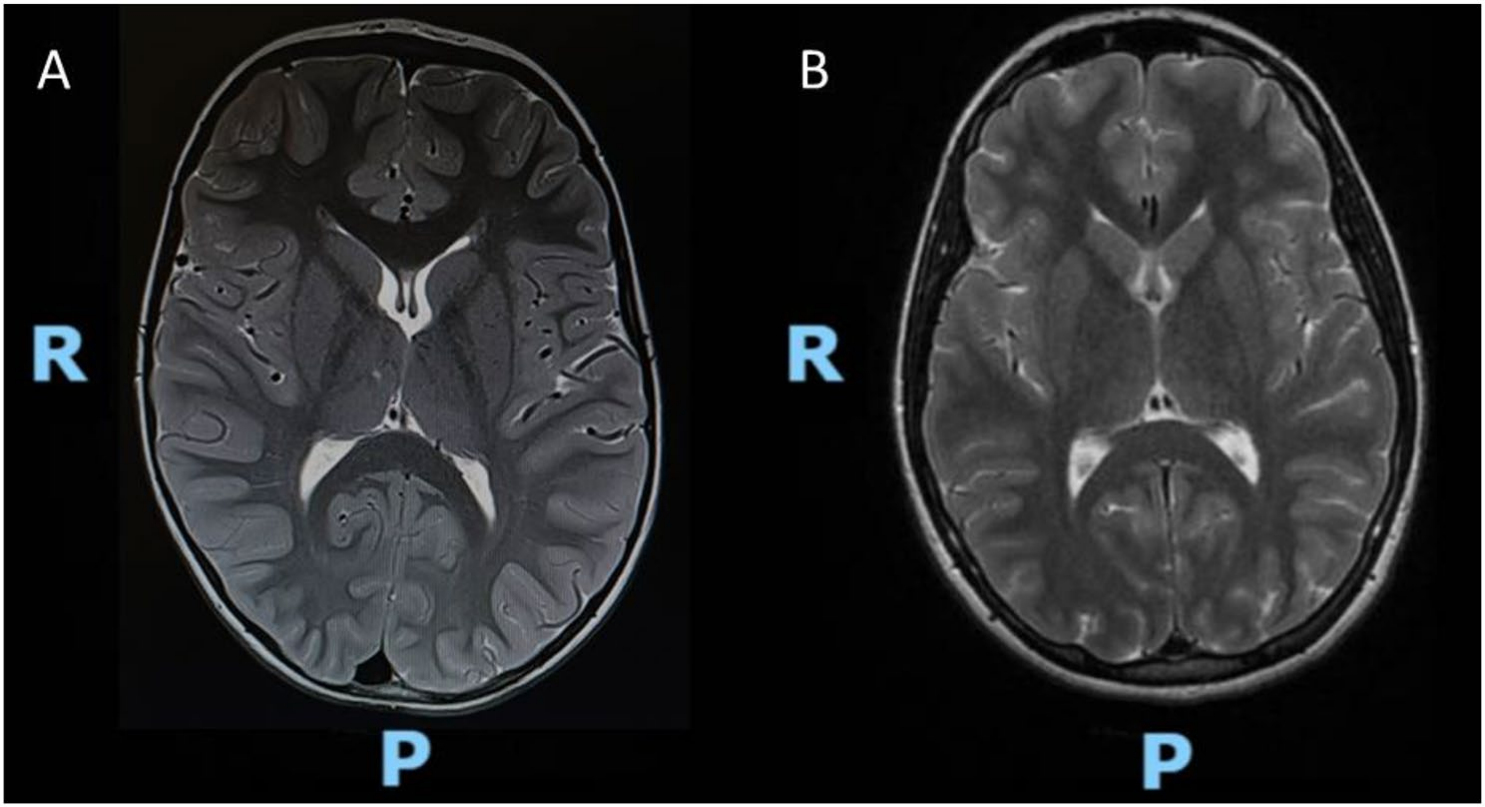
**A**-**B**. Brain MRI of the present patient at the onset during status epilepticus and at the age of 11 y.o. (T2-weighted images). **A**. The initial MRI at five years old showed mild hyperintensity in peri-trigonal white matter on T2-weighted sequences. **B**. This finding subsequently resolved on the follow-up MRI at eleven years old. Legend: R=right side; P= posterior side

At 5 years old she was admitted to our Hospital in Catania for convulsive status epilepticus and respiratory failure. Electroencephalography (EEG) showed “*bilateral synchronous spike-and-wave complexes with some multifocal features*”, typical of generalized epilepsy. (Fig. 1A) Brain Magnetic Resonance Imaging (MRI) was performed, showing mild accentuation of peri-trigonal white matter signal suggestive of congenital chronic hypoxia sequelae (Fig. 2A). The patient primarily experiences generalized tonic-clonic seizures, sometimes preceded by a focal onset with impaired awareness, based on the 2017 ILAE classification [5].

At 8 years old, learning disorder (dyslexia, dyscalculia and dysgraphia) was diagnosed after neuro-psychiatric evaluation according to the Diagnostic and Statistical Manual of Mental Disorders Fifth Edition, Text Revision (DSM-5-TR), but Wechsler Intelligent Scale for Children (WISC-IV v.4; Wechsler, 2003) evaluation were within the normal range for age. Problem-solving was also inadequate, sleep disturbances and motor clumsiness were also noted.

At the age of 11, the MRI pattern was no longer detected (Fig. 2B).

During the last follow up, at 12 years old, EEG showed “*instable*,* polymorphic*,* medium-voltage background activity dominated by bilateral theta rhythm. Slow wave and spike-and-wave complexes are recorded over the centro-parieto-temporal regions of both hemispheres and over the vertex regions”* (Fig. 1B). Electrical abnormalities in the left occipital leads were reported and she started Levetiracetam as anticonvulsant therapy (starting with 10 mg/kg/day, progressive dose increase up to 25 mg/kg/day divided in two doses) with a good control of seizures.

However, sometimes epileptic episodes occurred despite the therapy, predominantly characterized by generalized tonic-clonic seizures, loss of consciousness, and upward deviation of the eyeballs, followed by hypotonia, lasting for a few minutes. Sometimes, seizures presented only with hypotonia, loss of consciousness, and a fixed gaze, without clonic movements.

The general physical and neurological examination did not show any noteworthy signs; no nystagmus or strabismus, normal cerebellar tests, good muscle tone and strength. Ophthalmological and cardiological examination were normal, such as abdomen ultrasound.

The last neuropsychiatric evaluation performed at the age of 12 showed persistence of non-progressive motor clumsiness, sleep disorders, and previously identified learning disorders (multiple spelling errors during writing and mental and written calculation; reading aloud not functional for comprehension). The patient’s motor coordination difficulties have been noted since around 8 years of age, mainly becoming more problematic with tasks that demand fine motor skills. Additionally, memory and comprehension difficulties, relational disorders with difficulties in peer relationships, and low frustation and tolerance were reported IQ 70.

There is no evidence of deterioration over time, but these ongoing challenges add to the complexity of her overall neurodevelopmental status.

Below, we provide a fuller account of her WISC-IV subscale findings at age 12, along with a brief comparison to her evaluation at age 8:

### Cognitive testing at age 12

- The patient was re-assessed using the WISC-IV. Her Full-Scale IQ (FSIQ) was 70, placing her in the borderline range. Subscale results were as follows (all scaled to a mean of 100, SD of 15):

- ** ○Verbal Comprehension Index (VCI): 72**
 -Exhibited weaknesses in vocabulary breadth and verbal abstract reasoning (e.g., difficulty explaining word meanings, forming analogies).

- ** ○Perceptual Reasoning Index (PRI): 75**
 -Showed mild deficits in spatial reasoning and problem-solving, particularly on tasks requiring pattern construction or visual analysis under time constraints.

- ** ○Working memory index (WMI): 67**
 -Marked difficulty recalling sequences of digits and manipulating basic numerical information, suggesting an increased cognitive load leads to errors.

- ** ○Processing speed index (PSI): 68**
 -Displayed slowed performance on tasks requiring rapid visual scanning and motor output, such as coding and symbol search.

### Evidence of sectorial deterioration since age 8

- At age 8, the patient underwent a more informal cognitive assessment (initially guided by clinical observation and school-based psychoeducational testing). Although a precise FSIQ was not formally reported at that time, her general performance was estimated to be within the low-average range (roughly 80–85). This is notably higher than the borderline FSIQ obtained at age 12.
- The most striking differences emerged in Working Memory and Processing Speed, areas critical for keeping pace with increasing academic demands and more complex social interactions. In particular, her teachers reported that tasks once manageable at age 8 (e.g., following multi-step instructions, performing short mental calculations) became progressively more challenging by age 12.

### Motor performance and coordination

- Although the WISC-IV does not directly measure motor performance, concurrent testing with a standard motor coordination assessment (e.g., the Bruininks-Oseretsky Test of Motor Proficiency or Movement Assessment Battery for Children) revealed a mild decline in fine-motor speed and coordination compared to her prior evaluation. Tasks such as handwriting, manipulating small objects, or performing timed manual dexterity drills have grown more difficult since age 8, suggesting a sectorial deterioration or, at least, a failure to keep pace with age-level expectations.

### Interpretation and clinical relevance

- These observations indicate that the patient’s borderline intellectual functioning has become more pronounced as academic, social, and motor demands increased from age 8 to age 12.
- Rather than a dramatic or generalized cognitive decline, our data suggest that her deficits have become more evident in specific domains (working memory, processing speed, and fine motor coordination), reflecting an interplay between her underlying neurodevelopmental vulnerabilities and the heightened challenges of early adolescence.

Despite a good compliance in therapy, seizures persist, occurring approximately once a month, characterized by generalized hypertonia, loss of consciousness, and a fixed gaze, lasting for a few minutes, with subsequent spontaneous resolution.

### Genetic testing and data analysis

After obtaining informed consent from the proband’s parents, peripheral blood samples were collected from the affected child and her healthy parents to perform familial trio-Whole Exome Sequencing (trio-WES).

Exome enrichment was performed using Kapa Hypercapture Roche and sequenced with NovaSeq 6000 (Illumina, San Diego, CA, USA). Paired-end sequence reads were aligned to the human genome reference (hg19) with the Burrows-Wheeler Aligner (BWA MEM) software and duplicate reads removed using Picard (http://picard.sourceforge.net). Variant Calling were performed using an inhouse pipeline based on GATK v.4.3 tools (https://software.broadinstitute.org/gatk/).

The data were filtered and annotated using GEMINI v0.19.1 and the Variant Effect Predictor (VEP). Variants with a minor allele frequency (MAF) less than 0.1% were retained by comparison with public databases, including the 1000 Genomes Project, the Genome Aggregation Database (gnomAD), and the NHLBI Exome Sequencing Project (ESP) 6500. These variants were prioritized under assumptions of dominant or recessive inheritance patterns and based on their predicted functional effects from CADD, PolyPhen-2, and SIFT.

Variants were classified according to to the American College of Medical Genetics Genomics (ACMG) and the Association for Clinical Genomic Science (ACGS) recommendations (update 2023; uk-practice-guidelines-for-variant-classification-v1-2023) [5], with Varsome (https://varsome.com/) and Franklin (https://franklin.genoox.com/).

Sequenced data were visualized using Integrative Genomics Viewer (IGV). The forward primer (5’ CAGGGTTGGAGCACTGCCTCAT 3’) and reverse primer (5’ TCACCGTCTCGTAGTCTCGCAG 3’) were utilized for both amplification and Sanger sequencing of a 290 bp amplicon containing the *PPP5C* variant. The result interpretation was also implemented by searching relevant data from scientific literature.

### Protein mutational prediction

The 3D structure (PDB entry: 1A17) related to the human protein PPP5C (protein ID: P53041) was used to investigate in the Uniprot database (http://www.uniprot.org/) and RCSB Protein DataBase (PDB) (www.RCSB.org.it) the effect of the genetic variation within the protein and possible patogenic implications into the stability and activity.

### WES analysis

**Fig. 3:**
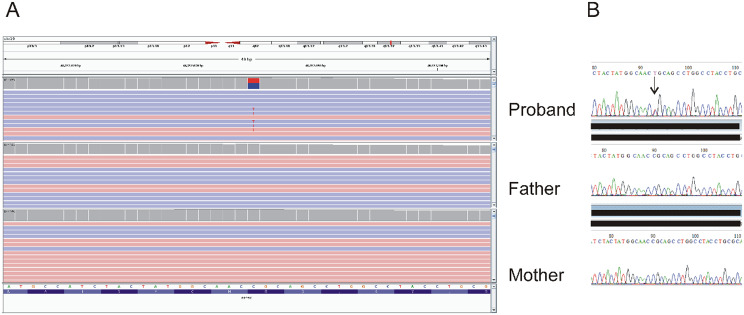
**A**-**B**. Identification and confirmation of the heterozygous c.202 C > T variant in *PPP5C* within the familial trio. (**A**) IGV screenshot displaying the heterozygous c.202 C > T variant in the *PPP5C* gene in the proband, absent in both parents. (**B**) Sanger sequencing electropherogram confirming the variant detected by WES. The black arrow indicates the position of the variant in the proband’s sequence

WES analysis revealed as a unique candidate a heterozygous, de novo missense variant in the *PPP5C* gene (NM_006247.4: c.202 C > T: p.Arg68Cys) encoding for a serine/threonine phosphatase involved in various cellular processes, including cell growth, stress response, and DeoxyriboNucleic Acid (DNA) damage repair. The variant was visually inspected using IGV and its segregation was technically confirmed by Sanger sequencing on the trio (Fig. 3A-B). The missense variant was classified as likely pathogenic based on criteria PM2 (variant not observed in control populations), PS2 (de novo variant, paternity and maternity confirmed), and PP3 (in silico computational predictions).

**Fig. 4:**
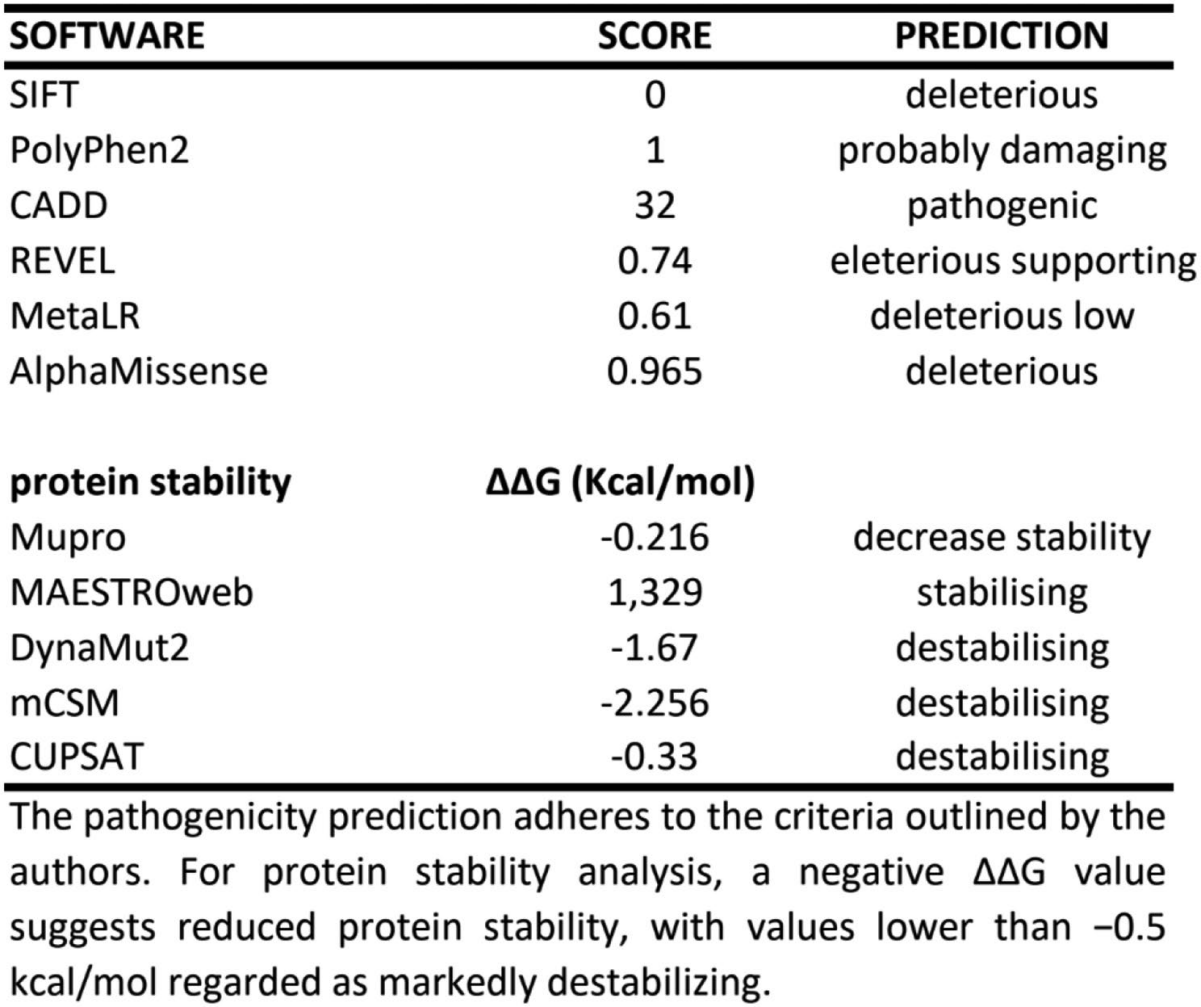
In silico DNA and protein predictions for the pathogenicity of the *PPP5C* p.Arg68Cys variant

Our in silico analysis revealed that the argine residue at position 68 of *PPP5C* is highly conserved across species (MutationTaster) and that multiple prediction tools indicated that the variant is likely deleterious and destabilizing at the protein level (Fig. 4).

Moreover, AlphaFold protein structure database (https://alphafold.ebi.ac.uk) also suggested that the missense variant is likely to disrupt the alpha helix, as indicated by the per-residue model confidence score (pLDDT) between 0 and 100, with a measured score (pLDDT > 90). This alteration could affect the superhelical structures of the tetratricopeptide repeat (TPR) domain, which is involved in protein-protein interactions [6].

The protein structure analysis performed by Uniprot and PDB databases allowed us to reconstruct the potential effect of the variant detected in our proband on the stability and function of PPP5C. We integrated protein and genetic data related to the present variant (Arg68Cys), but also to the variant (Ala47Thr) studied by Fielder et al. [1].

**Fig. 5:**
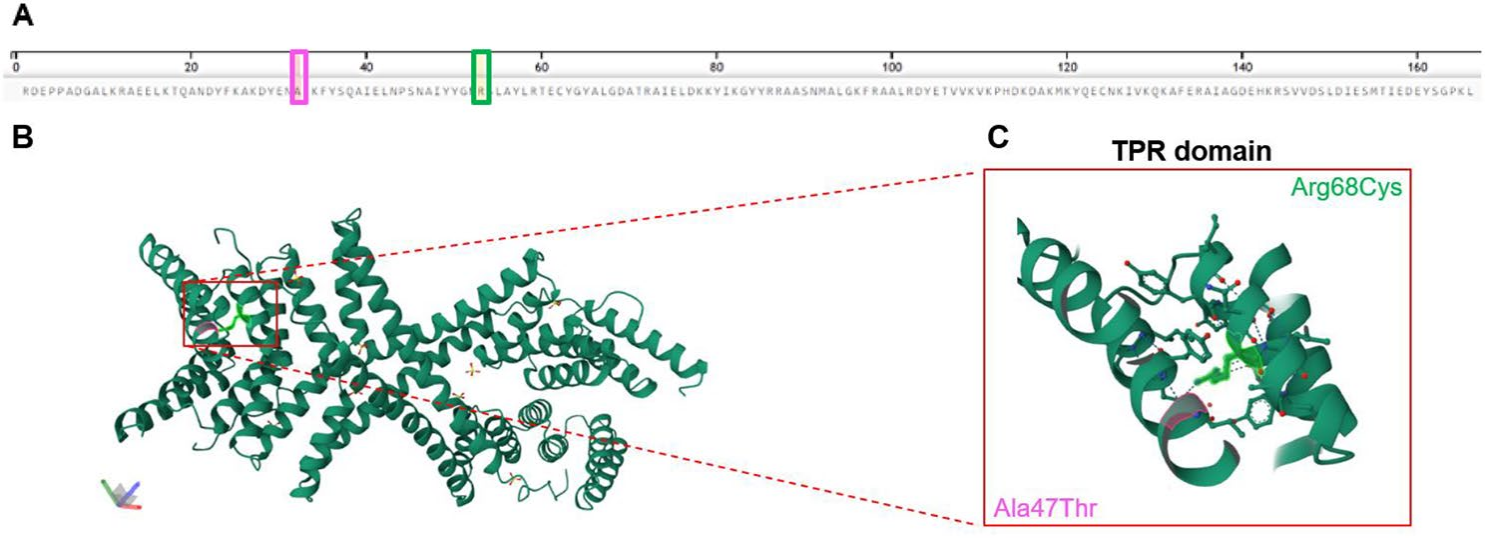
**A**-**C**. Graphical representation of the primary and tertiary structure of PPP5C showing the Arg68Cys variant (present case) and the Ala47Thr variant [1] (**A**) Visualization of the native amino acid sequence of the PPP5C protein (PBD entry 1A17) from RCSB PDB Database (https://www.rcsb.org/), covering residues 16 to 181. The position and the amino acid of the two different variants are highlighted: *Arg68Cys* in the present case (green box) and *Ala47Thr* in the study of Fielder et al. [1] (pink box). (**B**) The location in the 3D protein structure of both variants are marked with a red rectangle. Each variant is coloured in pink and green, respectively. (**C**) A zoom-in of the helical structure of the TPR domain where the two variants occur

Our findings were illustrated in Fig. 5A-C in view to assess the clinical relevance of both variants, which alter the domain function of helix and TPR repeat.

## Discussion

DEE are an heterogeneous group of disorders characterized by early-onset, severe epileptic seizures and EEG abnormalities on a background of developmental impairment that tends to worsen as a consequence of epilepsy [7]. Usually, DEE manifests during early infantile or childhood age with a significant impact on the child’s neurodevelopment and may result from both non-genetic and genetic aetiologies. Approximately 900 genes have been linked to DEE, with patients predominantly carrying pathogenic *de novo* variants [8].

Recently, advancements in next-generation sequencing have led to the discovery of new genes in patients with DEE of previously unknown etiology.

Our case report illustrated a pediatric patient with epilepsy, learning disorders, and a *de novo* heterozygous missense variant (c.202 C > T: *p.Arg68Cys*) in the *PPP5C* gene identified by WES. This variant was not previously described nor associated with learning disorders in the literature. The onset of seizures in this case was convulsive status epilepticus with respiratory failure, and anticonvulsant therapy initiated at the age of 5 led to a significant improvement in seizures. Additionally, the patient was diagnosed with learning disorders at the age of 8, a condition not previously linked to *PPP5C* gene mutations.

*PPP5C* modulates various signaling pathways by dephosphorylating target proteins, impacting cellular stress responses and interacting with hormone receptors, particularly within the MAPK and glucocorticoid pathways. Dysregulation of *PPP5C* has been linked to cancer progression and other disorders due to its critical role in cell cycle control and stress response regulation. Interestingly, a recent discovery report of a de novo missense variant in *PPP5C* in an eight-year-old girl with microcephaly, epilepsy, and developmental delays suggests a potential role for this gene in DEE [1]. However, this patient was later diagnosed with a Valyl-TRNA Synthetase 1 (VARS1) related condition, according to ClinVar variation (ClinVar ID: VCV001704329.2), making the contribution of this variant to the phenotype unclear. A comparison of clinical and genetic findings between our child and the case reported by Fielder et al. [1] was illustrated in the following Table 1.

**Table 1:** Clinical and genetic findings reported in the present case and compared with fielder et al. [1]

	Present patient	Fielder et al. 2022 [1]
Sex	Female	Female
Age at epilepsy onset	5 years old	12–16 months old
Age at diagnosis	12 years old	6 years old
EEG	Instable, polymorphic, medium-voltage background activity dominated by bilateral theta rhythm at 12 years old. Slow wave and spike-and-wave complexes are recorded over the centro-parieto-temporal regions of both hemispheres and over the vertex regions.	Recurrent spike and slow-wave activity independently and multifocal spikes.
Type of seizure	Status epilepticus, generalized tonic-clonic seizures.	Generalized seizures, including tonic clonic and drop seizures that are medically refractory.
Brain MRI	Mild accentuation of peri-trigonal white matter signal suggestive of congenital chronic hypoxia sequelae (5 years old), improved during follow-up brain MRI at 11 years old.	Reduced cerebral white matter volume + slightly incomplete myelination (11 months old), improved during follow-up brain MRI at 4 years old.
*PPP5C* gene variant	Heterozygous, de novo missense variant: NM_006247.4: c.202 C > T: *p.Arg68Cys*.	Heterozygous, de novo missense variant NM_006247.3: c.139G > A: *p.Ala47Thr.*
Neurodevelopmental	Normal achievement of developmental milestones.	Rolled over at 8–9 months old with therapies, sat up after age one year, currently unable to walk independently and unable to talk.
Physical exam	Normal.	Microcephaly (-5.5/6 D.S.) and synophrys.
Ophthalmologic exam	Normal.	Pendular nystagmus and moderate myopia.
Others	Learning disorders (inadequate problem-solving, dyscalculia, dysgraphia, dyslexia), sleep disturbances, motor clumsiness.	Failure to thrive and anaemia.

According to clinical features shown in Table 1, our case turned out to be phenotypically milder than Fielder’s case. A brain MRI at four years of age showed reduced cerebral white matter volume, while seizures, which began at 12 to 16 months of age, were refractory to treatment. On the other hand, our patient presented with a later onset of seizures (5 years), without microcephaly or other abnormalities on physical examination, with normal acquisition of neurodevelopmental milestones, unlike the other patient previously reported. Brain MRI initially showed mild accentuation of peri-trigonal white matter signal, suggestive of congenital chronic hypoxia sequelae, with a non-progressive course and signs of resolution in subsequent follow-ups. These MRI changes are less likely to be due to established hypoxic-ischemic injury and may instead reflect transient or subtle inflammatory or metabolic processes. No reduction of cerebral white matter volume was reported, differently from the other girl. Her cognitive and motor profiles align more closely with a ”*broad-spectrum neurodevelopmental disorder*” than a simple “learning disorder” alone. While we do not see classic signs of regressive encephalopathy, the sectorial deterioration in targeted domains underscores her increasing academic and social difficulties.

These findings, with a later onset of symptoms, suggesting a potentially milder phenotype. Despite the different phenotypic features observed, a potential link between genetic pathogenic variants in *PPP5C* and neurological developmental disorders could be suggested. Interestingly, data of Test for Association with De Novo Alterations (TADA) collected in the Simons Foundation Autism Research Initiative (SFARI) (https://www.sfari.org/resource/simons-simplex-collection/) and in the Autism Sequencing Consortium (https://asc.broadinstitute.org/) identified *PPP5C* gene as a candidate gene of autism spectrum disorders (ASD) with a false discovery rate between 0.05 and 0.1 [9, 10]. This finding reinforces the involvement of *PPP5C* as a player in neurological pathways underlying *PPP5C*-related phenotypes. In addition, persistent motor clumsiness may signify mild cerebellar or extrapyramidal involvement, potentially relevant to the *PPP5C* variant.

Given the current lack of a clearly defined clinical phenotype associated with *PPP5C* variants, our case may provide some valuable insights into the spectrum of phenotypic manifestations linked to this gene. While our findings provide valuable insights, this study has several limitations. The experimental animal model using *C. elegans* developed by Fielder et al. [1] suggests a possible link between *PPP5C* and a neurological phenotype; however, the dual diagnosis in their patient complicates direct clinical comparisons with our case. Additionally, we acknowledge that interpreting the functional roles of novel candidate variants—particularly missense or non-coding regulatory variants—requires careful consideration. It should be noted that this study lacks in vivo and in vitro functional analysis to support the pathogenicity of the detected *PPP5C* variant. Considering these limitations, the potential role of this gene in milder forms of epilepsy not associated with encephalopathy should be explored, alongside its possible involvement in neurodevelopmental disorders such as learning difficulties and borderline intellectual functioning.

Therefore, it is crucial to investigate whether other cases have been described, but not yet recognized probably due to the under-reporting cases. This could ultimately aid in the identification and management of similar cases in the future, emphasizing the need for ongoing research and data collection in this emerging area of study.

## Conclusion

The *PPP5C* gene, as a member of the serine/threonine protein phosphatases family, is widely expressed across human tissues, although its precise physiological functions remain not fully understood. To date, despite the identification of two cases with pathogenic variants in the *PPP5C* gene, including the girl reported in this study, a decisive association between a specific clinical phenotype to these genotypes cannot yet be established. Thus, the potential role of pathogenic variants in the *PPP5C* gene in epilepsy and neurodevelopmental disorders warrants significant attention. The re-analysis of cases not solved or unfairly overlooked as clinically-not informative reports would be crucial to confirm this potential association and to elucidate the contribution of *PPP5C* to neurological condition.

## Data Availability

Data is provided within the manuscript.

## Abbreviations

ACGS: Association for Clinical Genomic Science
ACMG: American College of Medical Genetics Genomics
ASD: Autism Spectrum Disorders
BWA MEM: Burrows-Wheeler Aligner
CNS: Central nervous systems
DEE: Developmental and epileptic encephalopathy
DNA: DeoxyriboNucleic Acid
EEG: Electroencephalography
ESP: Exome Sequencing Project
gnomAD: Genome Aggregation Database
IGV: Integrative Genomics Viewer
MAF: Minor Allele Frequency
MRI: Magnetic Resonance Imaging
PDB: Protein DataBase
PRI: Perceptual Reasoning Index
pLDDT: Per-residue model confidence score
PPP5C: Protein Phosphatase 5 Catalytic Subunit
PSI: Processing Speed Index
SFARI: Simons Foundation Autism Research Initiative
TADA: Test for Association with De Novo Alterations
TPR: Tetratricopeptide repeat
VARS1: Valyl-TRNA Synthetase 1
VCI: Verbal Comprehension Index
VEP: Variant Effect Predictor
WES: Whole Exome Sequencing
WISC-IV: Wechsler Intelligent Scale for Children v.4
WMI: Working Memory Index

## References

[1] Fielder SM, Rosenfeld JA, Burrage LC, Emrick L, Lalani S, Attali R et al (2022) Functional analysis of a novel de Novo variant in PPP5C associated with microcephaly, seizures, and developmental delay. Mol Genet Metab 136(1):65–73 35361529 10.1016/j.ymgme.2022.03.007 PMC10200280

[2] Hamilton CL, Abney KA, Vasauskas AA, Alexeyev M, Li N, Honkanen RE et al (2018) Serine/threonine phosphatase 5 (PP5C/PPP5C) regulates the ISOC channel through a PP5C-FKBP51 axis. Pulm Circ 8(1):2045893217753156 29283027 10.1177/2045893217753156 PMC6018905

[3] Hinds TD, Sánchez ER (2008) Protein phosphatase 5. Int J Biochem Cell Biol 40(11):2358–2362 17951098 10.1016/j.biocel.2007.08.010 PMC2551559

[4] Zhang H, Zhang Q, Tu J, You Q, Wang L (2023) Dual function of protein phosphatase 5 (PPP5C): an emerging therapeutic target for drug discovery. Eur J Med Chem 254:115350 37054560 10.1016/j.ejmech.2023.115350

[5] Scheffer IE, Berkovic S, Capovilla G, Connolly MB, French J, Guilhoto L et al (2017) ILAE classification of the epilepsies: position paper of the ILAE commission for classification and terminology. Epilepsia 58(4):512–521 28276062 10.1111/epi.13709 PMC5386840

[6] Das AK, Cohen PW, Barford D (1998) The structure of the tetratricopeptide repeats of protein phosphatase 5: implications for TPR-mediated protein-protein interactions. EMBO J 17(5):1192–1199 9482716 10.1093/emboj/17.5.1192 PMC1170467

[7] Guerrini R, Conti V, Mantegazza M, Balestrini S, Galanopoulou AS, Benfenati F (2023) Developmental and epileptic encephalopathies: from genetic heterogeneity to phenotypic continuum. Physiol Rev 103(1):433–513 35951482 10.1152/physrev.00063.2021 PMC9576177

[8] Scheffer IE, Zuberi S, Mefford HC, Guerrini R, McTague A (2024) Author correction: developmental and epileptic encephalopathies. Nat Rev Dis Primer 10(1):1–1 10.1038/s41572-024-00558-2 39271712

[9] Iossifov I, O’Roak BJ, Sanders SJ, Ronemus M, Krumm N, Levy D et al (2014) The contribution of de Novo coding mutations to autism spectrum disorder. Nature 515(7526):216–221 25363768 10.1038/nature13908 PMC4313871

[10] Satterstrom FK, Kosmicki JA, Wang J, Breen MS, De Rubeis S, An JY et al (2020) Large-Scale exome sequencing study implicates both developmental and functional changes in the neurobiology of autism. Cell 180(3):568–584e23 31981491 10.1016/j.cell.2019.12.036 PMC7250485

